# Preparation, Structural Characterization, and Synergistic Hypoglycemic Effect of Jujube Polysaccharide–Polyphenol Complex

**DOI:** 10.3390/foods15030552

**Published:** 2026-02-04

**Authors:** Zheng Ye, Wenjing Wang, Yumei Li, Qiaoshuang Lu, Chun Yang

**Affiliations:** 1 Shanxi Institute for Functional Food, Shanxi Agricultural University, Taiyuan 030001, China; yezheng@sxau.edu.cn; 2 College of Food Science and Engineering, Shanxi Agricultural University, Jinzhong 030801, China; 20233070@stu.sxau.edu.cn (W.W.); 15682681908@163.com (Y.L.); z20223035@stu.sxau.edu.cn (Q.L.)

**Keywords:** polysaccharide, polyphenol, polysaccharide-polyphenol complex, hypoglycemic, identification, interaction

## Abstract

Type 2 diabetes mellitus (T2DM) is a globally prevalent chronic metabolic disorder that poses severe public health risks. Synthetic hypoglycemic agents are susceptible to inducing adverse reactions, thus driving the development of natural, safe and highly effective plant-derived hypoglycemic active compounds as a research hotspot. Inhibiting the activity of α-glucosidase and α-amylase represents an effective strategy to regulate postprandial blood glucose levels. This study investigated the synergistic hypoglycemic activity of a composite (PS-PP) formed by polysaccharide (PS) and polyphenols (PP) from Ziziphus jujuba Mill. cv. Muzao and elucidated the structural basis underlying this synergistic effect. First, MPS and MPP were isolated and purified, followed by the in vitro assembly to prepare PS-PP. The hypoglycemic activities of MPS, MPP and MPS-PP were evaluated via in vitro enzyme inhibition assays, while structural characterization was conducted using GPC-MALLS, FT-IR and SEM techniques. Results demonstrated that PS-PP exerted the strongest activity under optimal conditions (0.75 mg/mL concentration, pH 4.0, 1:2 mass ratio), with IC_50_ values of 1.14 μg/mL and 0.82 μg/mL against the two enzymes, which were superior to those of polysaccharides (15.10 and 36.06 μg/mL) and polyphenols (1.18 and 46.24 μg/mL). Structural analysis revealed that the interaction between PS and PP was primarily mediated by hydrogen bonds. PS-PP exhibited significant differences from single-component compounds in molecular weight distribution, functional group binding and surface morphology. These structural alterations were identified as the key factors contributing to its enhanced hypoglycemic efficacy. This study clarifies the synergistic hypoglycemic mechanism of MPP-PS and lays a scientific foundation for the development of natural hypoglycemic preparations and functional foods.

## 1. Introduction

Diabetes mellitus (DM) is one of the most prevalent metabolic disorders worldwide, imposing a substantial burden on the global economy and healthcare systems [1]. According to the latest statistics from the International Diabetes Federation (IDF), approximately 585 million adults aged 20–79 years were living with diabetes worldwide in 2025, among which type 2 diabetes mellitus (T2DM) accounted for about 90%. It is characterized by hyperglycemia resulting from insulin resistance or impaired insulin secretion [2]. Currently, long-term administration of commonly used clinical hypoglycemic agents (metformin, sulfonylureas) may induce adverse reactions such as gastrointestinal discomfort, hypoglycemia, and drug resistance, which not only reduce therapeutic efficacy but also potentially increase the risk of complications [3]. In contrast, phytochemicals derived from natural plant sources offer advantages such as low cytotoxicity and high safety profiles. Consequently, recent research has focused on isolating bioactive plant constituents—such as polysaccharides and polyphenols—that exhibit hypoglycemic properties for T2DM prevention and management [4,5]. Notably, *Muzao*, predominantly cultivated in Shanxi Province, ranks among China’s top ten jujube cultivars. The polysaccharide and polyphenol constituents of this leading variety have garnered considerable interest due to their mild hypoglycemic effects [6,7].

*Muzao*, a botanical species belonging to the Rhamnaceae family, has been utilized in Traditional Chinese Medicine for millennia and is often referred to as the “fruit of life” compared with other consumable fruits [8,9]. It is extensively employed as a phytotherapeutic agent for its laxative, anti-constipation, and diuretic properties in Asian medical practices, particularly in China [10]. Pharmacological research has identified jujube as a rich source of phenolic phytochemicals, with its polysaccharide compounds demonstrating regulatory effects on glycemic control, enhancement of insulin sensitivity, and attenuation of mitochondrial dysfunction [11,12].

Polysaccharides are extensively distributed across plant, animal, and microbial systems [13,14,15], exhibiting a range of bioactivities including antioxidant, hypoglycemic, and immunomodulatory effects [16]. Peng et al. [17] demonstrated that conjugation with phenolic compounds significantly enhances the structural integrity and functional efficacy of lotus root polysaccharides. To augment the bioactivity of polysaccharides, the formation of polysaccharide–polyphenol complexes presents a promising approach. Polyphenols, as secondary plant metabolites [18], play critical roles in the prophylaxis of chronic diseases such as vascular disorders, diabetes mellitus, and neurodegenerative conditions [19]. Due to the environmental susceptibility of the active phenolic hydroxyl groups in polyphenols—leading to oxidation, degradation, or isomerization—stability concerns arise [20,21]. Hydrophilic polysaccharides can enhance the solubility of polyphenols in aqueous media, safeguarding them from environmental degradation and improving their stability within the gastrointestinal tract [22,23]. These findings substantiate the synergistic interaction between polysaccharides and polyphenols.

The interaction between polysaccharides and polyphenols not only enhances the bioactivity of polyphenols but also modifies the structure of polysaccharides, thereby expanding their applicability in fields such as food technology and medicine [20]. Enzyme inhibition of α-glucosidase and α-amylase serves as a promising strategy for controlling postprandial hyperglycemia in diabetic management [24]. Song et al. [25] examined both in vivo and in vitro hypoglycemic effects of a garlic polysaccharide (GPs)-Cr(III) chelate, demonstrating significantly enhanced inhibitory activity against α-glucosidase compared to GPs alone. Other investigations have shown that the hesperidin-Cu(II) complex exhibits markedly superior inhibitory effects on α-glucosidase and α-amylase activities relative to hesperidin monomers [24]. Kim et al. [26] explored the physicochemical processing-induced interactions between plant proteins and polyphenols, revealing that polyphenol incorporation induces conformational alterations in protein structures, thereby potentiating their biofunctional properties. Although numerous studies have reported polysaccharides and polyphenols in various jujube cultivars, as well as the hypoglycemic potential of polysaccharide–polyphenol (PS-PP) complexes from diverse plant and fruit matrices, the MPS-PP complexes differ significantly in structure and function from previously reported jujube or fruit- and vegetable-derived counterparts. First, compared with common cultivars (e.g., *Dongzao*, *Junzao*), *Muzao* has a distinctive polyphenol profile (flavonoids accounting for 42.9% of total identified polyphenols) and specific polysaccharide monosaccharide molar ratio (arabinose: galactose: rhamnose = 35.68:31.00:13.58), endowing its PS-PP complexes with a unique structural basis. Second, unlike existing studies on jujube PS-PP complexes that only focus on simple physical mixing, the present study investigated the effects of various factors on the preparation of the MPS-PP complex. This preparation process induced more stable intermolecular interactions (hydrogen bonding and hydrophobic interactions) and a stronger synergistic hypoglycemic activity compared with physically mixed complexes. Third, in contrast to previous studies assessing only single carbohydrate-hydrolyzing enzyme inhibition, this study systematically investigated the dual inhibitory effects of MPS-PP complexes on α-glucosidase and α-amylase, confirmed their superior synergistic activity over individual components and physical mixtures, and elucidated associated structural changes.

The aim of this study was to investigate the optimization of the preparation process, hypoglycemic activity, and structural characterization of the polysaccharide–polyphenol complex derived from *Muzao*. In vitro hypoglycemic assays guided the process for the conjugation of *Muzao* polysaccharides with polyphenols. By analyzing the differences in bioactivity and structural configurations between the complexes and their individual constituents, the synergistic effects were confirmed, and the structural modifications contributing to enhanced bioactivity were elucidated. Overall, this work seeks to exploit *Muzao*’s therapeutic potential as a source of high-value bioactive compounds, address the limitations of poor polyphenol stability and bioavailability, and provide a theoretical foundation for developing novel natural antidiabetic agents and functional foods.

## 2. Materials and Methods

### 2.1. Instruments and Reagents

#### 2.1.1. Instruments

Fourier Infrared Spectrometer was obtained from Beijing Jingke Ruida Technology Co. (Beijing, China); JSM-IT800 Scanning Electron Microscope (SEM) from Suzhou Safetech Electronics Co. (Suzhou, China); Gel Permeation Chromatography System Agilent 1260 from Shanghai Huishi Instrument Co. (Shanghai, China); Ion Chromatograph ICS-5000 from Wuxi Hastelloy Technology Co. (Wuxi, China); High-Performance Liquid Chromatograph UltiMate 3000 from Ivana Xinyang Instrument Equipment Co. (Xinyang, China); and OPTILAB T-rEX, Oscillometric Detector, and DAWN HELEOS-II Laser Light Scattering Detector from Wyatt Technology, Goleta, CA, USA. Details of commonly used instruments are provided in the Supplementary Material Table S1.

#### 2.1.2. Reagents

Anhydrous ethanol, concentrated hydrochloric acid, methanol, chloroform, and n-butanol were obtained from Tianjin Zhiyuan Chemical Reagent Co. (Tianjin, China). Phenol was sourced from Tianjin Damao Chemical Reagent Factory. Soluble starch, anhydrous sodium carbonate, sodium chloride, and sodium hydroxide were purchased from Tianjin Yongda Chemical Reagent Co. (Tianjin, China). Additionally, Tris-HCl buffer solution (pH 8.2), anhydrous D-glucose, DNS reagent, α-glucosidase, α-amylase, acarbose, folinol, gallic acid standard, and p-nitrophenyl-α-D-glucopyranoside (PNPG) were acquired from Shanghai Yuanye Bio (Shanghai, China).

### 2.2. Raw Material Pre-Treatment

*Muzao*: Mature *Muzao* were sourced from Shanxi Qi Kou Hong Agricultural Technology Co., Ltd., located in Lüliang City, Shanxi Province, China. After selection, the fruits underwent cleaning, pitting, secondary washing, pulping, and freeze-drying. The dried *Muzao* were then ground into powder using a high-speed homogenizer, sieved through a 60-mesh screen, packaged into resealable pouches, and stored at −20 °C for subsequent analytical procedures.

DEAE cellulose DE-52: The DE-52 anion exchange resin powder was immersed in distilled water overnight to ensure complete swelling. After impurity removal by vacuum filtration, the resin was transferred to a 0.5 M HCl solution and soaked for two hours. It was then rinsed with distilled water until the pH was neutral or above 4, followed by air-drying. Subsequently, the resin was immersed in 0.5 M NaOH for 2 h, rinsed with distilled water until neutral pH was achieved, and air-dried again.

Macroporous resin: At room temperature, different types of resin were soaked in 95% ethanol for 24 h. They were then rinsed with distilled water until odorless and clear, with all powder particles removed, followed by immersion in 5% HCl for 12 h. After rinsing with distilled water to neutral pH, the resin was finally soaked in 5% NaOH for 12 h. Pretreatment was completed by rinsing with distilled water and checking the pH with pH test paper. A 2–5 cm layer of 95% ethanol solution was maintained on the resin surface for subsequent processing [27].

### 2.3. Extraction of Muzao Polysaccharides and Polyphenols

#### 2.3.1. Standard Curve of Polysaccharides

Polysaccharide content was determined using the phenol–sulfuric acid method [28]. Absorbance was measured at 490 nm with a UV-Vis spectrophotometer (Beijing Puxi General Instrument Co., Beijing, China). A standard curve was constructed using glucose standards to obtain the regression equation.

(1) $$y=0.087+0.0056x,R^{2}=0.9993$$

#### 2.3.2. Polyphenol Standard Curve

The Folin–Ciocalteu spectrophotometric method [29] was used to measure the total phenolic content in *Muzao* with some modifications. We mixed 20 μL of the extract with 100 μL of Folin–Ciocalteu, incubated it for 5 min, then added 80 μL of sodium carbonate solution and 50 μL of ultrapure water. We incubated the mixture at room temperature and protected it from direct light for one hour. We measured the absorbance at 765 nm using a microplate reader. We calculated the total phenolic content with a gallic acid standard curve and determined the polyphenol concentration using the corresponding regression equation.

(2) $$y=0.092+0.0048x,R^{2}=0.9996$$

#### 2.3.3. Extraction of Polysaccharides

Following the polysaccharide extraction method described by Ji et al. [30] with appropriate modifications, *Muzao* powder was mixed at a 1:30 material-to-liquid ratio, and the mixture was incubated in a 70 °C water bath for 50 min. It was then centrifuged at 5000 rpm for 20 min to separate the supernatant, which was collected and concentrated under vacuum at 60 °C. Next, one-quarter volume of Sevage reagent (chloroform:n-butanol, 4:1) was added to the concentrated extract after rotary evaporation [31], and this step was repeated twice. After 48 h of dialysis, four volumes of anhydrous ethanol were added to the dialysate, which was then stored overnight at 4 °C. The resulting precipitate was collected by centrifugation at 5000 rpm for 10 min, redissolved, and freeze-dried under vacuum to obtain crude *Muzao* polysaccharides.

#### 2.3.4. Extraction of Polyphenol

Using the method outlined by Chmelová et al. [32] with slight modifications, 80% methanol was used at a 1:40 material-to-solvent ratio for ultrasonic-assisted extraction at 50 °C for 40 min. The sample was extracted repeatedly once then subjected to vacuum concentration at 45 °C, freeze-drying, and storage at −20 °C for subsequent analysis.

### 2.4. Process of Polysaccharide Purification

#### Cellulose DE-52 Purification

*Muzao* polysaccharides were dissolved in distilled water to prepare a solution at the desired concentration. This solution was centrifuged at 5000 rpm for 15 min, and the clear supernatant was filtered through a 0.45 μm membrane to prepare the loading solution. Using a dropper, the entire loading solution was carefully applied to a DEAE cellulose-52 anion exchange resin chromatography column [33]. After equilibrating the system for 10 min, sequential elution was performed with 0.1–0.5 mol/L NaCl solutions and distilled water at a flow rate of 1.0 mL/min. Fractions of 20 mL were collected at each elution step. The phenol–sulfuric acid method was used to monitor the process by measuring absorbance at 490 nm [28]. Fractions with higher polysaccharide content, indicated by absorption peaks, were pooled. This combined solution was then concentrated, dialyzed, freeze-dried under vacuum, and stored at –20 °C for future use.

### 2.5. Process of Polysaccharide Purification Using Macroporous Resins

#### 2.5.1. Resin Screening Method

Since the adsorption performance of macroporous resins is closely related to their polarity, surface properties and pore structure, we selected six types of macroporous resins (D101, HPD-100, AB-8, ADS-17, DA201, and XAD2) with different polarities, specific surface areas and average pore sizes for comparative screening. The method described by Yang et al. [34] was adopted with minor adjustments. Precisely weigh 0.5 g of various wet resins and add 20 mL of *Muzao* polyphenol extract at 0.25 mg/mL. The samples were then placed in a constant-temperature shaker at 25 °C and 120 rpm for 24 h for static adsorption. The absorbance of polyphenols was measured, and the static adsorption rate was calculated using Equation (3). After adsorption equilibrium was reached, the resins were transferred to conical flasks containing an equal volume of 95% ethanol solution. Desorption was performed for 24 h with 20 mL of 95% ethanol under the same shaking conditions. The desorption rate was then determined according to Equation (4).

(3) $$Adsorption\;rate\;\left(\%\right)=\frac{C_{0}-C_{1}}{C_{0}}\times 100$$

(4) $$Desorption\;rate\;\left(\%\right)=\frac{C_{2}}{C_{0}-C_{1}}\times 100$$

In this formula, C_0_, C_1_, and C_2_ represent the concentrations of the sample solution, the adsorption filtrate, and the desorption collection solution, respectively, measured in milligrams per milliliter (mg/mL).

#### 2.5.2. Static Adsorption and Desorption Experiments

We measured five pre-treated D101 resin samples of 0.5 g each. Sample solutions were prepared at concentrations of 0.1, 0.25, 0.50, 0.75, and 1.0 mg/mL; pH levels were adjusted to 2.0, 3.0, 4.0, 5.0, and 6.0; and desorption solution concentrations were set at 40%, 50%, 60%, 70%, and 80%. These samples were then incubated in a constant-temperature shaker. Absorbance was measured at intervals to calculate the adsorption and desorption rates of D101 resin for *Muzao* polyphenols, enabling the identification of optimal loading conditions.

#### 2.5.3. Dynamic Adsorption and Desorption Experiments

Building on the static experiments, we used the wet column loading technique to investigate how varying loading flow rates (0.50, 0.75, 1.00, 1.50, and 1.75 mL/min), loading volumes (100–400 mL), and elution volumes (150–550 mL) affect adsorption and desorption efficiency. Eluate samples were collected every 20 mL to measure the absorbance of *Muzao* polyphenols, calculate adsorption and desorption rates, generate leakage curves, and determine the optimal dynamic conditions.

### 2.6. In Vitro Antidiabetic Activity

#### 2.6.1. Assay of α-Glucosidase Inhibition Activity

Using a modified protocol by Li et al. [35], 400 µL of pH 6.8 phosphate buffer, 200 µL of sample solution, and 200 µL of α-glucosidase solution (5.2 U/mL) were mixed. The mixture was incubated in a 37 °C water bath for 15 min. Next, 400 µL of PNPG solution (2.5 mmol/L) was added, and the reaction proceeded at 37 °C for an additional 15 min. The reaction was terminated by adding 5 mL of 0.1 mol/L sodium carbonate solution. An acarbose-positive control group was set up concurrently, and absorbance was measured at 405 nm. An interference control group (PBS replacing enzyme) was established to eliminate spectrophotometric interference; a blank control group (PBS replacing both sample and enzyme) to correct the background absorbance of the reaction system; a negative control group (reaction buffer replacing sample) to ensure maximum enzymatic catalytic activity under the experimental conditions. The final results were calculated using the following formula:

(5) $$\alpha \text{-}glucosidase\;inhibition\;rate\;\left(\%\right)=\left(1-\frac{A_{2}-A_{0}}{A_{1}-A_{C}}\right)\times 100$$

In the formula, A_1_ represents the sample group, A_2_ represents the experimental group, and A_C_ represents the control group.

#### 2.6.2. Assay of α-Amylase Inhibition Activity Method

Based on the method of Wu et al. [36] with some modifications, 500 μL of sample solution and 500 μL of α-amylase solution (5.0 U/mL) were mixed and incubated at 37 °C for 30 min. Next, 200 μL of 1% soluble starch solution was added, and the reaction proceeded for 20 min. Then, 1 mL of DNS chromogen was added, and the mixture was heated in a boiling water bath for 10 min. It was immediately cooled to room temperature in an ice bath, and the volume was adjusted to 25 mL. An acarbose-positive control group was set up concurrently, and absorbance was measured at 540 nm. An interference control group (PBS replacing enzyme) was established to eliminate spectrophotometric interference; a blank control group (PBS replacing both sample and enzyme) to correct the background absorbance of the reaction system; a negative control group (reaction buffer replacing sample) to ensure maximum enzymatic catalytic activity under the experimental conditions. The final results were calculated using the following formula:

(6) $$\alpha \text{-}amylase\;inhibition\;rate\;\left(\%\right)=\left(1-\frac{B_{2}-B_{0}}{B_{1}-B_{C}}\right)\times 100$$

In the formula, B_1_ represents the sample group, B_2_ represents the experimental group, and B_C_ represents the control group.

### 2.7. Preparation of Polysaccharide–Polyphenol Complex

Drawing on insights from prior research and experimental experience with *Muzao* polysaccharides and polyphenols [37,38,39], we investigated the effects of concentration, pH, and mass ratio on the composite. Purified polysaccharides and polyphenols were formulated into composite solutions at concentrations of 0.05, 0.10, 0.25, 0.50, and 0.75 mg/mL, pH values of 2.0, 3.0, 4.0, 5.0, and 6.0, and mass ratios of 3:2, 2:1, 1:1, 1:2, and 2:3. Following the procedures in Section 2.6.1 and Section 2.6.2, the inhibitory rates of α-glucosidase and α-amylase were determined to identify the optimal preparation conditions for the composite.

### 2.8. Structural Characterization

#### 2.8.1. Polyphenol Components 

Column conditions: Chromatographic column: ACQUITY UPLC HSS T3 (1.8 µm, 2.1 mm × 100 mm); Mobile phase: Phase A (0.1% formic acid in water), Phase B (0.1% formic acid in acetonitrile); Elution gradient: 0–1.5 min, A/B (95:5, %/%); 2.5 min, A/B (95:5, %/%); 14 min, A/B (60:40, %/%); 24–27 min, A/B (5:95, %/%); 27.1–30 min, A/B (95:5, %/%); Column temperature: 40 °C; Injection volume: 4 μL [40,41].

Mass spectrometry conditions: First-stage mass spectrometry data acquisition range: 50–1200 *m*/*z*; collision energy: 30 eV; acquisition time: 15 s per cycle. Electrospray ionization (ESI) parameters: ionization gas pressure, 60 psi; auxiliary gas pressure, 60 psi; curtain gas pressure, 35 psi; temperature, 550 °C; spray voltage, 5500 V (positive ion mode) or –4500 V (negative ion mode).

#### 2.8.2. Monosaccharide Composition of Polysaccharides

An appropriate amount of polysaccharide sample was weighed, 1 mL of 2 M TFA solution added, and the mixture heated at 121 °C for 2 h. The solution was then nitrogen-purged and blow-dried, cleaned with 99.99% methanol and blow-dried again, repeating the methanol cleaning step 2–3 times. Sterile water was added for dissolution, and the solution transferred to a chromatographic vial for analysis. Accurately weighed standard samples were diluted with water to prepare a 10 mg/mL stock solution. Appropriate volumes of the stock solution were then mixed to prepare standard mixtures with maximum concentrations of 60 μg/mL, 50 μg/mL, and 40 μg/mL. These gradients constituted the serial standard samples required for instrumental analysis [42,43].

Analysis was conducted on a Thermo ICS 5000+ ion chromatography system (Thermo Fisher Scientific, Waltham, MA, USA) equipped with a Dionex™ CarboPac™ PA20 column (150 mm × 3 mm, 10 µm) and a 5 µL injection volume. Mobile phases were A (H_2_O), B (0.1 M NaOH), and C (0.1 M NaOH + 0.2 M NaAc), at a flow rate of 0.5 mL/min. Column temperature was maintained at 30 °C, with the elution gradient programmed as follows: 0–25 min: A/B/C (95:5:0, *v*/*v*); 26 min: A/B/C (85:5:10, *v*/*v*); 42 min: A/B/C (85:5:10, *v*/*v*); 42.1 min: A/B/C (60:0:40, *v*/*v*); 52 min: A/B/C (60:40:0, *v*/*v*); 52.1 min: A/B/C (95:5:0, *v*/*v*); 60 min: A/B/C (95:5:0, *v*/*v*). The monosaccharide composition of the polysaccharide sample solution was determined via this system.

#### 2.8.3. Determination of the Molecular Weight of Polyphenols

The analysis was performed on an Agilent Technologies 1260 high-performance liquid chromatography (HPLC) system (Agilent, Santa Clara, CA, USA) equipped with a G1362A RID detector. Chromatographic conditions were as follows: Column: Waters Ultrahydrogel (300 mm × 7.8 mm); mobile phase: 0.1 mol/L sodium nitrate aqueous solution; flow rate: 1 mL/min; column temperature: 40 °C.

#### 2.8.4. Determination of the Molecular Weight of Polysaccharides and Polysaccharide–Polyphenol Complexes

The sample was dissolved in 0.1 M NaNO_3_ aqueous solution (0.02% *w*/*w* NaN_3_) to a final concentration of 1 mg/mL, then filtered through a 0.45 µm filter and prepared for use. The chromatographic system comprised a U 3000 liquid chromatography system (Thermo, USA), an Optilab T-rEX differential detector (Wyatt Technology, CA, USA), and a DAWN HELEOS II multi-angle laser light scattering system (Wyatt Technology, CA, USA). Chromatographic columns and elution conditions: Ohpak SB-805 HQ (300 mm × 8 mm) and Ohpak SB-803 HQ (300 mm × 8 mm) gel exclusion columns were connected in series [44,45]. Column temperature was maintained at 45 °C with an injection volume of 100 μL; isocratic elution was performed with Mobile phase A (0.02% NaN_3_, 0.1 M NaNO_3_) at a flow rate of 0.6 mL/min for 75 min. Comprehensive molecular characterization of the sample was achieved by combining chromatographic separation, differential detection and light scattering data acquisition.

#### 2.8.5. Fourier Infrared Spectroscopy (FT-IR)

In a dry environment, jujube polysaccharides, polyphenols, and polysaccharide–polyphenol complexes were mixed with potassium bromide at a 1:100 ratio, pressed into tablets using a tablet press, and scanned with a Fourier transform infrared spectrometer over a wavelength range of 4000–500 cm^−1^ at a resolution of 4 cm^−1^ and with 64 scans [46].

#### 2.8.6. Electron Microscopy (SEM)

Appropriate amounts of dried polysaccharides, polyphenols, and polysaccharide–polyphenol complexes were separately taken, attached to a conductive carbon film with double-sided adhesive, and placed in the sample chamber of an ion sputtering instrument for gold sputtering [47]. After gold sputtering, the samples were transferred to a scanning electron microscope (SEM), and their micromorphological features were observed at different magnifications.

### 2.9. Data Analysis and Processing

All experiments were conducted with three biological replicates. Experimental data were collated in Microsoft Excel 2024, and data normality was verified by the Shapiro–Wilk test, with data deemed normally distributed at *p* > 0.05. Subsequent statistical analysis was performed via one-way analysis of variance (ANOVA) in the General Linear Model (GLM) module of IBM SPSS Statistics 26. The significance level was set at *p* < 0.05, and all results were expressed as mean ± standard deviation (SD). Graphical visualization was performed using OriginPro 2024.

## 3. Results

### 3.1. Polysaccharide and Polyphenol Standard Curves

The standard calibration curves for polysaccharide and polyphenol quantification were moved to the Supplementary Materials (Figure S1). As shown in Figure S1, both curves met the requirements for quantitative analysis. Based on these curves, the contents of polysaccharides (63.56%) and polyphenols (27.46%) in the purified sample were calculated to be 6.75 and 3.51 times higher than those in the crude extract (9.41% and 7.83%), respectively.

### 3.2. Process of Polyphenol Purification Using Macroporous Resin

#### 3.2.1. Resin Screening

Adsorption and desorption rates were used as core evaluation criteria for resin screening, with high values for both ensuring effective enrichment and efficient elution of polyphenols. As shown in Table 1, the six resins exhibited significant differences in adsorption and desorption behavior (*p* ≤ 0.05). D101, HPD100, and AB-8 resins showed the highest adsorption and desorption rates for MPP (86.75%, 82.54%, 80.01% and 94.41%, 88.71%, 86.71%, respectively), with D101 resin performing the best. ADS-17 resin had the lowest adsorption and desorption rates among all tested resins. Considering both resin properties and adsorption/desorption rates, D101 resin was selected for the subsequent purification process.

#### 3.2.2. Static Adsorption and Desorption of D101 Resin

**Figure 1:**
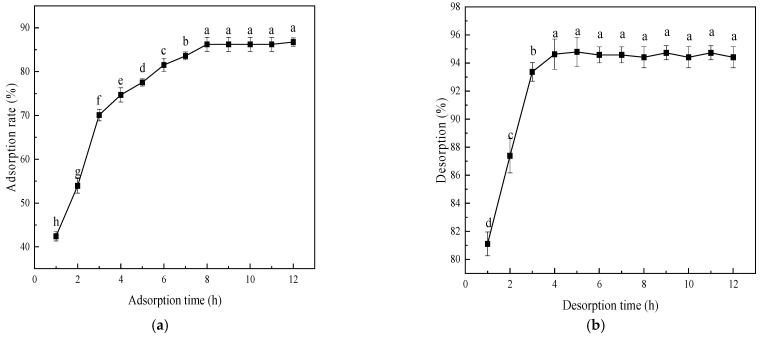
Static adsorption (**a**) and desorption (**b**) kinetic curves of *Muzao* polyphenols on D101 macroporous resin. Values are expressed as the mean ± SD (*n* = 3). Values labeled with different letters are significantly different based on Duncan’s multiple range test (*p* < 0.05).

Figure 1a shows the static adsorption kinetic curve of MPP on D101 resin. The curve reveals the adsorption rate rises with time until equilibrium, increasing rapidly in the initial 0–4 h, then gradually rising to a saturation of 86.75% at 4–8 h before stabilizing. Thus, the optimal adsorption duration was determined as 8 h.

Figure 1b shows the static desorption kinetic curve of MPP from D101 resin after adsorption equilibrium. The desorption rate displayed a parabolic trend, rising sharply over the first 0–3 h, increasing more slowly from 3 to 4 h and stabilizing at 94.30% after 4 h. Thus, 4 h was determined as the optimal desorption duration.

#### 3.2.3. Impact of Loading Concentration and pH on Adsorption Rate

**Figure 2:**
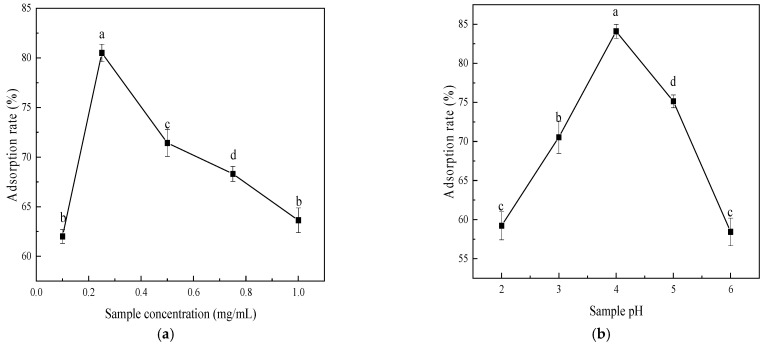
Effects of sample loading concentration (**a**) and pH (**b**) on the static adsorption of *Muzao* polyphenols by D101 macroporous resin. Values are expressed as the mean ± SD (*n* = 3). Values labeled with different letters are significantly different based on Duncan’s multiple range test (*p* < 0.05).

As shown in Figure 2a, the adsorption rate of D101 resin for MPP increased with rising sample concentration, peaking at 0.25 mg/mL, then decreasing with further increases in concentration. Thus, optimal adsorption performance is achieved at a loading concentration of 0.25 mg/mL.

The adsorption rate of MPP increased then decreased with increasing pH (Figure 2b). At pH below 4, the resin exhibited enhanced MPP adsorption capacity, reaching a peak adsorption rate of 84.09%. Thus, MPP adsorption should be performed under acidic conditions at pH 4.

#### 3.2.4. Effect of Desorption Solution Concentration on Desorption Rate

**Figure 3:**
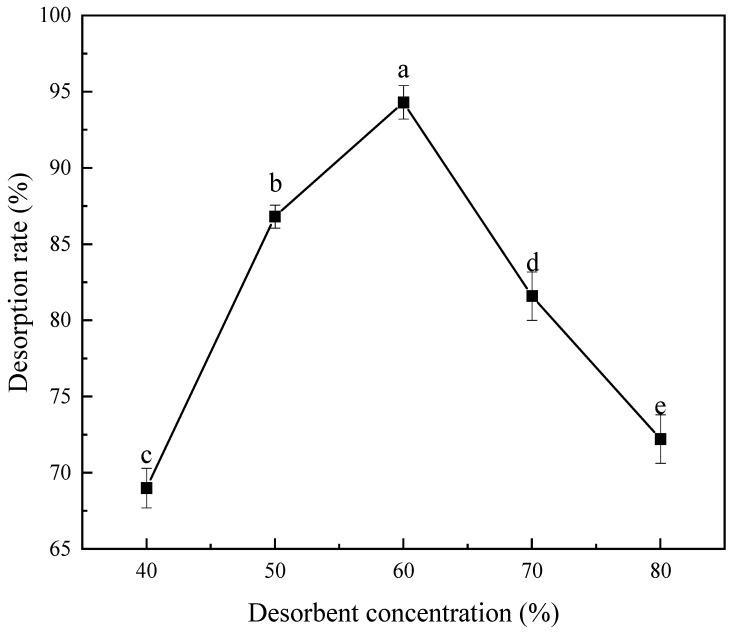
Effects of different ethanol eluent concentrations on the desorption rate of *Muzao* polyphenols from D101 macroporous resin. Values are expressed as the mean ± SD (*n* = 3). Values labeled with different letters are significantly different based on Duncan’s multiple range test (*p* < 0.05).

As shown in Figure 3, the MPP desorption rate of D101 resin increases initially then decreases with rising desorption solution concentration, peaking at 94.30% at 60% concentration. Based on this trend, 60% ethanol was identified as the optimal desorption solution concentration for MPP.

#### 3.2.5. Impact of Loading Volume and Desorption Volume on Adsorption/Desorption

**Figure 4:**
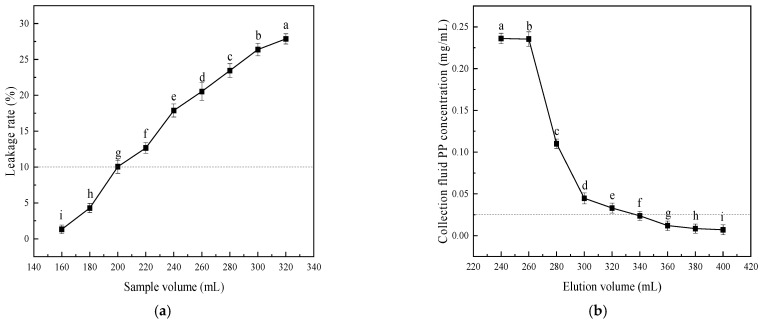
Effects of sample loading volume (**a**) and desorption volume (**b**) on adsorption and desorption rates of *Muzao* polyphenols by D101 macroporous resin. Dashed lines indicate that the adsorbent resin is close to or has reached its saturated adsorption capacity. Values are expressed as the mean ± SD (*n* = 3). Values labeled with different letters are significantly different based on Duncan’s multiple range test (*p* < 0.05).

The optimal loading volume (desorption capacity) is achieved when the polyphenol concentration in the eluate reaches 10% (1/10) of that in the loading solution (see Figure 4a). This critical point defines the resin’s polyphenol adsorption leakage threshold. At a loading volume of approximately 200 mL, the eluate’s polyphenol concentration reaches this threshold, confirming 200 mL as the optimal loading volume. Similarly, at a desorption volume of approximately 340 mL, the eluate’s polyphenol concentration drops to one-tenth of the loading concentration (Figure 4b), establishing 340 mL as the optimal desorption volume.

#### 3.2.6. The Impact of Sample Flow Rate on Adsorption Rate

**Figure 5:**
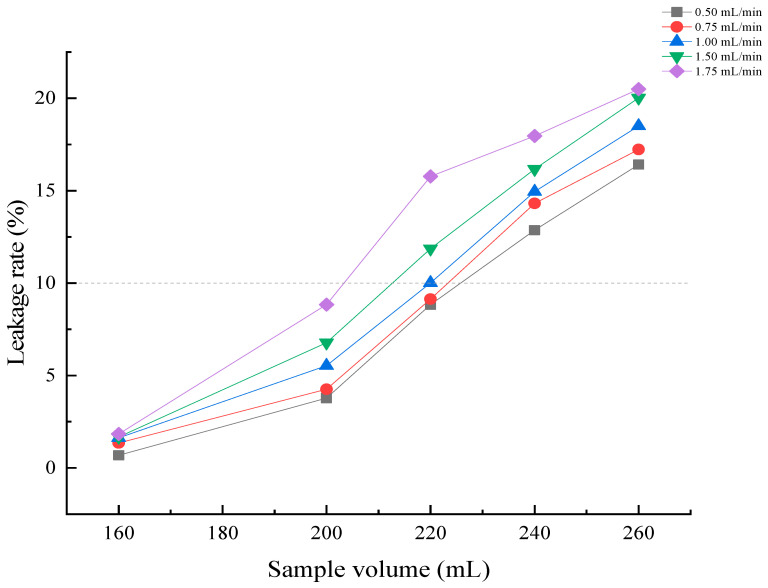
Effects of dynamic loading flow rate on the adsorption efficiency of *Muzao* polyphenols by D101 macroporous resin. The dashed line indicates that the adsorption resin is close to or has reached its saturated adsorption capacity.

As shown in Figure 5, the adsorption rate gradually declines or even plateaus with increasing loading volume at different flow rates, while polyphenol concentration in the residual liquid rises, causing premature emergence of leakage points. At an elution volume of 220 mL, eluate polyphenol concentration exceeds the leakage point concentration at 1.50 and 1.75 mL/min. In contrast, at 0.50 and 0.75 mL/min, eluate polyphenol concentration does not reach the leakage threshold even at higher elution volumes. Considering both adsorption efficiency and time cost, a flow rate of 1.0 mL/min was selected as the optimal loading flow rate for MPP.

### 3.3. In Vitro Hypoglycemic Activity

#### 3.3.1. The Effects of PS and PP Extraction and Purification on Hypoglycemic Activity

**Figure 6:**
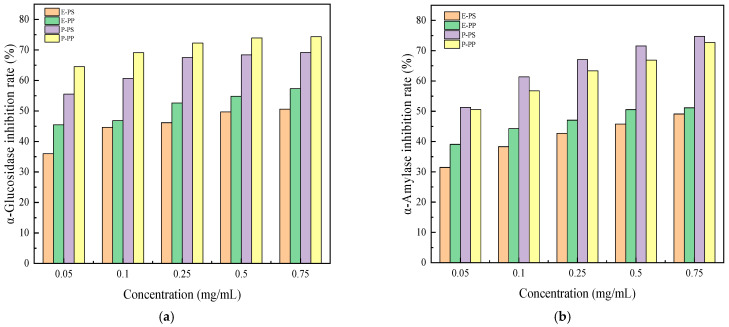
Inhibitory effects of crude extracts and purified products on α-glucosidase (**a**) and α-amylase (**b**) activities in vitro.

Type 2 diabetes mellitus (T2DM) is a chronic metabolic disorder characterized by impaired glucose and lipid metabolism. α-Glucosidase and α-amylase are key enzymes in carbohydrate metabolism, and their inhibition is closely associated with postprandial blood glucose regulation. Figure 6 depicts the inhibitory effects of extracted polysaccharides (E-PS), extracted polyphenols (E-PP), purified polysaccharides (P-PS), and purified polyphenols (P-PP) on α-glucosidase and α-amylase activity. Figure 6a,b show that at 0.05–0.75 mg/mL, E-PS/E-PP and P-PS/P-PP both exhibited concentration-dependent inhibition of these two enzymes. Purified polysaccharides and polyphenols had significantly higher inhibition rates (69.16%, 67.17%; 74.37%, 63.38%) than their extracted counterparts (50.60%, 49.10%; 57.31%, 51.15%). The IC_50_ values for α-glucosidase and α-amylase inhibition by extracted and purified PS and PP were 0.54828 mg/mL, 0.15812 mg/mL, 0.01510 mg/mL, and 0.00118 mg/mL and 0.86298 mg/mL, 0.48978 mg/mL, 0.03606 mg/mL, and 0.04624 mg/mL, respectively. These results confirm that high-purity polysaccharides and polyphenols exert superior inhibitory effects on α-glucosidase and α-amylase, thus prompting the preparation of purified polysaccharide–polyphenol complexes.

#### 3.3.2. The Effects of Purified PS, PP, and PS-PP Complex on Hypoglycemic Activity

**Figure 7:**
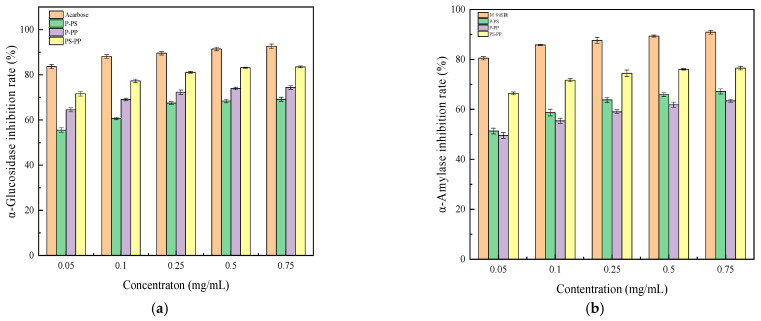
Inhibitory effects of purified *Muzao* polysaccharides (P-PS), polyphenols (P-PP) and their PS-PP complex on α-glucosidase (**a**) and α-amylase (**b**) activities in vitro. Values are expressed as the mean ± SD (*n* = 3).

As shown in Section 3.3.1, purified fractions exhibited higher inhibitory rates against both enzymes than their extracted counterparts. Figure 7 shows acarbose and purified PS, PP, and PS-PP exert concentration-dependent inhibition of α-glucosidase and α-amylase. The IC_50_ values of acarbose, PS, PP, and PS-PP against α-glucosidase were 0.00019 μg/mL, 0.01510 mg/mL, 0.00118 mg/mL, and 0.00113 mg/mL, respectively; the corresponding values against α-amylase were 0.00037 mg/mL, 0.03606 mg/mL, 0.04624 mg/mL, and 0.00082 mg/mL, respectively. The inhibitory potency against α-glucosidase and α-amylase was ranked as follows: α-glucosidase: acarbose > PS-PP > PP > PS; α-amylase: acarbose > PS-PP > PS > PP. At 0.75 mg/mL, the inhibitory rates of acarbose, PS, PP, and PS-PP on α-glucosidase were 92.65%, 69.16%, 74.37%, and 83.50%, respectively; the corresponding rates on α-amylase were 90.89%, 67.17%, 63.38%, and 76.52%, respectively. Although all three test substances had lower inhibitory effects than acarbose in the tested concentration range, the PS-PP complex outperformed individual PS and PP, highlighting considerable potential for the development of high-efficiency hypoglycemic agents.

### 3.4. Single-Factor Tests for PS-PP Preparation

#### 3.4.1. The Effects of Different Concentrations on Hypoglycemic Activity

**Figure 8:**
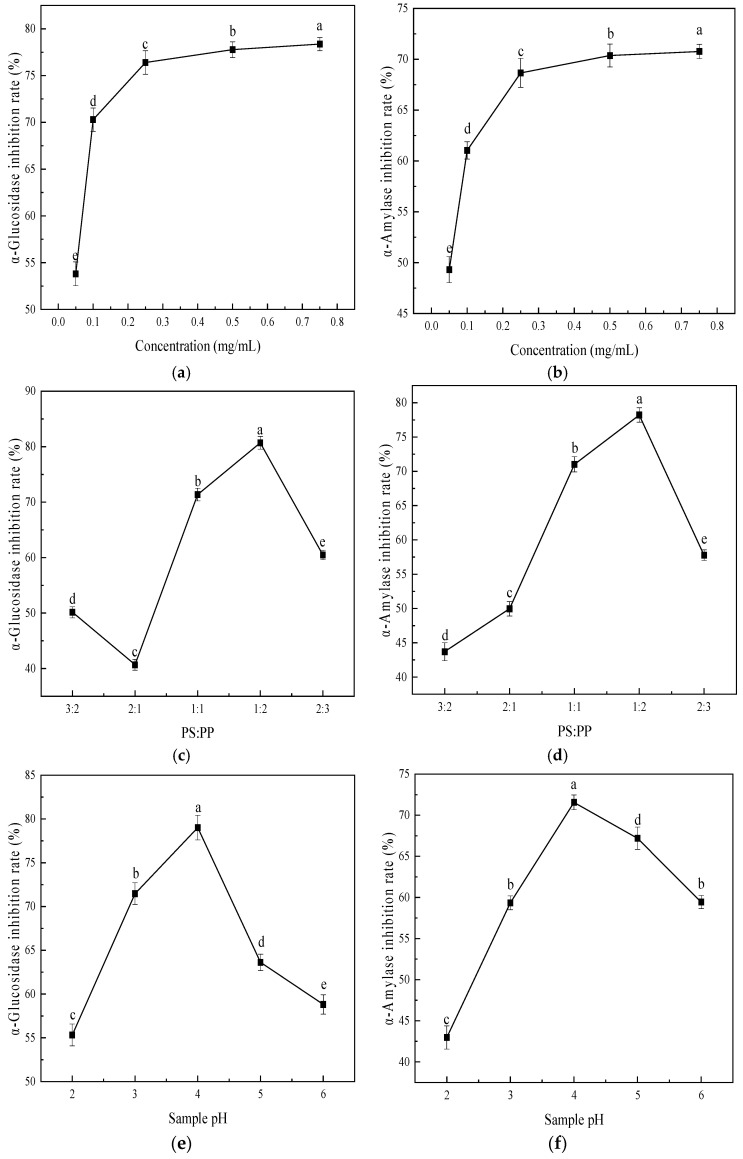
In vitro inhibitory effects of *Muzao* PS-PP complex on α-glucosidase and α-amylase activities: different concentrations (**a**,**b**), polysaccharide–polyphenol mass ratios (**c**,**d**), and reaction pH values (**e**,**f**). Values are expressed as the mean ± SD (*n* = 3). Values labeled with different letters are significantly different based on Duncan’s multiple range test (*p* < 0.05).

As shown in Figure 8a,b, the inhibitory effects of the complex on α-glucosidase and α-amylase activity showed parabolic trends with increasing concentration. In the range of 0.05–0.25 mg/mL, its inhibitory activity against both enzymes rose sharply, peaking at 78.59% and 77.37%, respectively. Above 0.25 mg/mL, the complex’s inhibitory activity reached near saturation, with only limited further improvement thereafter. Based on these trends, the optimal concentration of the polysaccharide–polyphenol complex was determined to be 0.75 mg/mL.

#### 3.4.2. The Effects of the Mass Ratio on Hypoglycemic Activity

The complex’s inhibitory activity showed a decrease, increase, then decrease with varying mass ratios (Figure 8c). At PS:PP ratios from 3:2 to 2:1, α-glucosidase inhibition declined sharply; from 2:1 to 1:2, the inhibition rate rose rapidly to a maximum of 81.00%, then declined thereafter. Figure 8d shows α-amylase inhibition by the complex increased then decreased at PS:PP ratios of 3:2–2:3. From 3:2 to 2:1, its inhibitory effect on α-amylase activity trended upward, peaking at 78.77% at a PS:PP ratio of 2:1. Thus, a PS:PP mass ratio of 1:2 was selected as the optimal ratio for the complex.

#### 3.4.3. The Effects of Different pH Levels on Hypoglycemic Activity

Figure 8e,f show that, at complex pH values of 2–6, the inhibitory effects on α-glucosidase and α-amylase activity increased then decreased with rising pH. Maximum inhibitory effects were observed at pH 4, reaching 79.01% and 71.56% for α-glucosidase and α-amylase, respectively. Consequently, the inhibitory effect was significantly weaker at pH 2. In summary, the polysaccharide–polyphenol complex should be prepared under acidic conditions at pH 4.

### 3.5. Polyphenol Components

**Figure 9:**
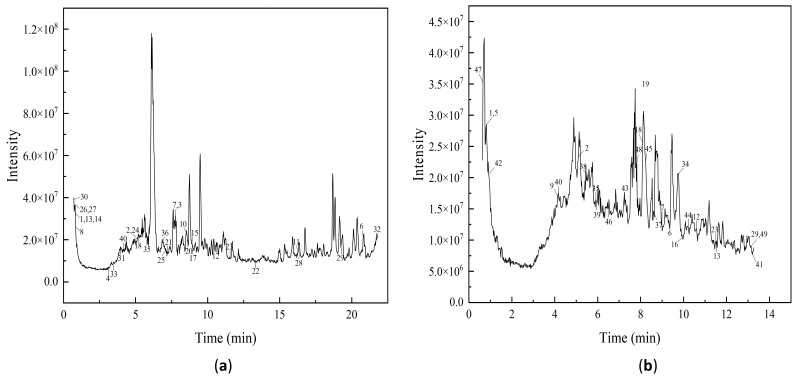
Positive ion mode (**a**) and negative ion mode (**b**) total ion chromatograms of purified *Muzao* polyphenols detected by LC-MSLC-MS. Note: The numbers labeled in the graphs represent substances with Table 2 serial numbers.

To better characterize the polyphenol composition of Muzao, untargeted metabolomic profiling was performed via LC-MS in both positive and negative ion modes. Metabolite identification was achieved by matching spectral data to relevant databases [45], followed by analysis of differential metabolic indicators based on peak alignment results. The main components of the purified extract were identified and listed in Table 2. Polyphenol components were identified through retention time matching, accurate molecular weight (*m*/*z*) alignment, and characteristic fragment ion matching with authentic standards (e.g., gallic acid, catechin, quercetin) and public mass spectral databases (MassBank, HMDB, NIST). A total of 49 phenolic compounds were identified under both ion modes. Figure 9 shows the total ion current profiles, with the *X*-axis representing retention time (min) and the *Y*-axis denoting ion current intensity (cps).

A total of 49 phenolic compounds were identified in purified MPP, classified into three major classes: 13 phenolic acids, 4 tannins, and 21 flavonoids (full details in Table 2). Flavonoids were the most abundant class (42.9%), representing the dominant polyphenols in *Muzao*, followed by phenolic acids (26.5%), while tannins accounted for the lowest proportion (8.2%). Among these, flavonols and characteristic phenolic acids were identified as the key bioactive components. Representative compounds for each class included gallic acid (phenolic acids), proanthocyanidin A1 and (-)-catechin gallate (tannins), and luteolin, quercetin and epicatechin (flavonoids). All these representative components are widely reported to exhibit anti-inflammatory, hypoglycemic and antioxidant activities.

Notably, three phenolic acids (gallic acid, caffeic acid, protocatechuic acid) previously reported in jujube polyphenols were not detected in this study. This observation is speculated to be associated with differences in detection techniques (LC-MS vs. HPLC) and sample pretreatment methods and may also reflect cultivar specificity or environmental variations in the polyphenol profile of *Muzao*.

### 3.6. The Monosaccharide Composition of Polysaccharides

**Figure 10:**
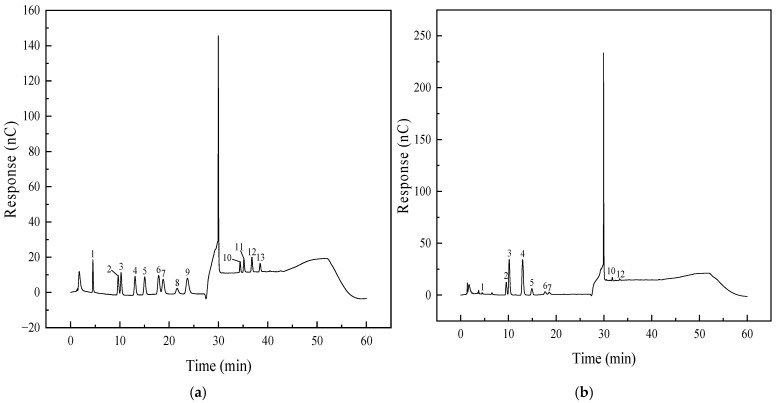
High-performance liquid chromatography profiles of monosaccharide standard mixture (**a**) and acid-hydrolyzed *Muzao* polysaccharide sample (**b**). Note: 1. Fucose; 2. Rhamnose; 3. Arabinose; 4. Galactose; 5. Glucose; 6. Xylose; 7. Mannose; 8. Fructose; 9. Ribose; 10. Galacturonic acid; 11. Glucuronide; 12. Mannuronic acid; 13. Guluronic acid.

Polysaccharide samples were hydrolyzed and their monosaccharide composition analyzed via ICS with an electrochemical detector. Figure 10 presents ion chromatograms of the mixed standard (a) and sample (b). Results show that Muzao polysaccharides comprise fucose (Fuc), rhamnose (Rha), arabinose (Ara), galactose (Gal), glucose (Glc), xylose (Xyl), mannose (Man), galacturonic acid (Gal-UA), and glucuronic acid (Glc-UA) (Figure 10b), with molar ratios of 0.81:13.58:35.68:31.00:5.71:3.19:2.81:5.33:1.89. Arabinose, galactose, and rhamnose are the major monosaccharide components of *Muzao* polysaccharides, while glucose, xylose, fucose, mannose, and uronic acid are present in minor amounts.

### 3.7. Molecular Weight of MPS, MPP and MPS-PP

**Figure 11:**
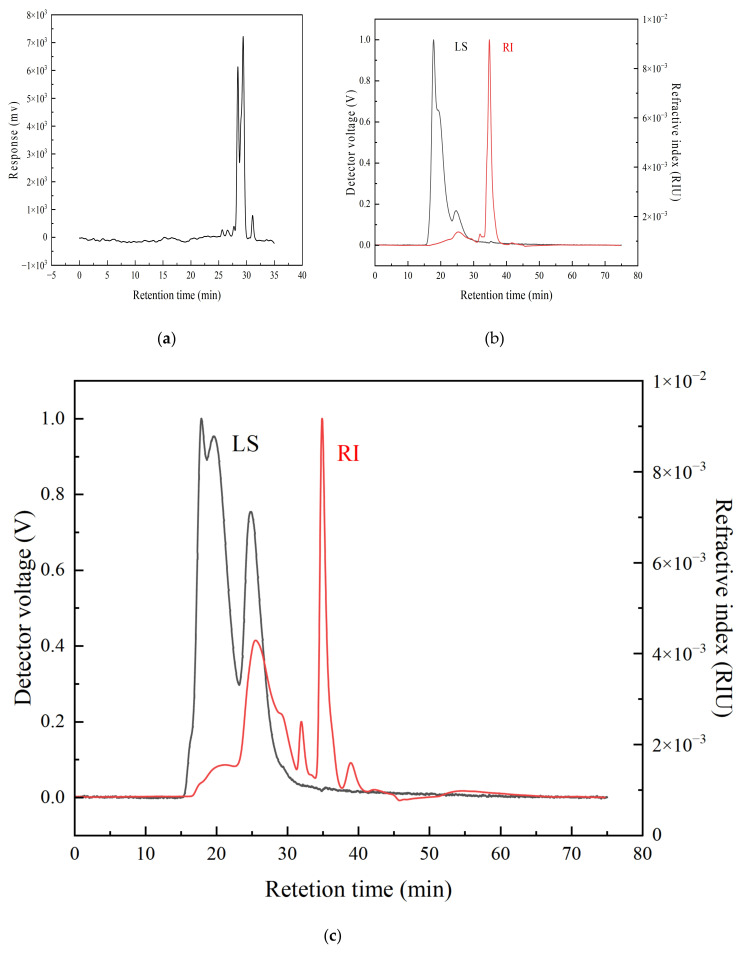
Molecular weight distribution of purified *Muzao* polyphenols (**a**), polysaccharides (**b**), and their PS-PP complex (**c**) determined by gel permeation chromatography.

The elution profile of MPS was obtained via GPC (Figure 11a), while those of MPS and the MPS-PP complex were acquired using GPC-RI-MALLS (Figure 11b,c). The polyphenol signal peaked at 25–30 min in Figure 11a, and the PS signal was detected at 34–40 min in Figure 11b. In Figure 11c, the PP and PS signals peaked at 20–32 min and 34–40 min, respectively, confirming the formation of the PS-PP complex. Two distinct regions in the LS curves of Figure 11b,c indicated the presence of components with molecular weights different from that of MPS. The Mw values of MPP, MPS, and PS-PP were 760 Da, 2.313 × 10^5^ Da, and 2.272 × 10^5^ Da, respectively, demonstrating a lower Mw for PS-PP compared with MPS.

### 3.8. Fourier Infrared Spectroscopy

**Figure 12:**
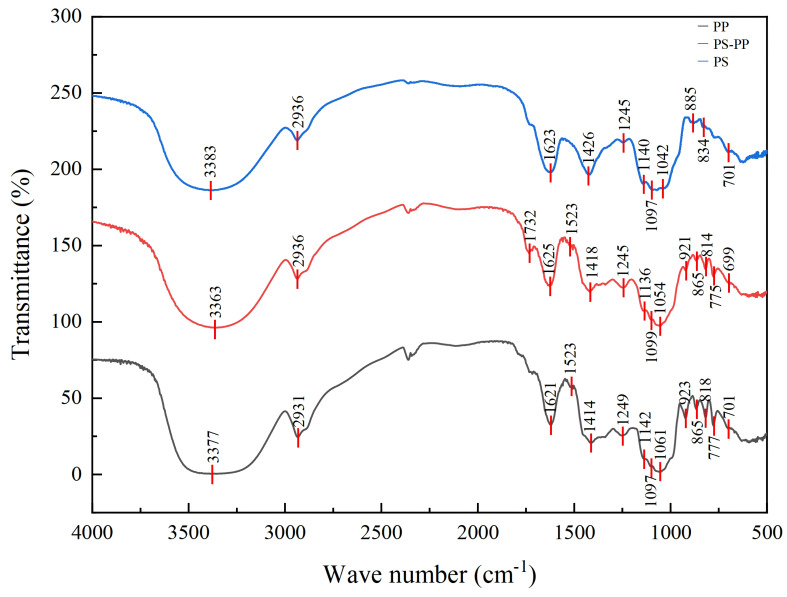
Fourier transform infrared (FT-IR) spectra of purified *Muzao* polysaccharides, polyphenols and their PS-PP complex.

To confirm MPS-PP complex formation, FT-IR spectra (4000–500 cm^−1^) of MPS, MPP monomers, and their conjugate were compared. Figure 12 presents FT-IR spectra of PS, PP, and PS-PP. The PS spectrum shows characteristic broad, intense absorption bands: O-H stretching vibrations (3383 cm^−1^); C-H stretching vibrations (2936, 1426, 1245 cm^−1^); C=O asymmetric stretching vibrations (1623 cm^−1^); pyranose characteristic peaks (C-O-C and C-O-H, 1200–1000 cm^−1^); α- and β-glycosidic bonds (885, 834 cm^−1^); and primary/secondary amines (701 cm^−1^). The PS-PP complex spectrum resembles PS but with key changes: the 3363 cm^−1^ O-H stretching band (from PP and sugar/hydrin) and enhanced intensity of the 2936 cm^−1^ C-H peak. Compared to PS, the complex has stronger absorption at 2936, 1732, and 865 cm^−1^; absent 885, 834 cm^−1^ peaks; distinct 1625, 1523 cm^−1^ peaks (benzene ring C=C); and 1418, 1245 cm^−1^ peaks (benzene or polysaccharide C-H). No new peaks were detected in 1200–1000 cm^−1^ for PS-PP, consistent with PS and MPP. MPP and PS-PP show multiple peaks at 750–900 cm^−1^, with PS-PP having an additional 699 cm^−1^ peak. Collectively, these peaks confirm the complex contains hydroxyl, carbonyl, amino sugar groups, polyphenols, and aromatic rings (typical PS/PP characteristics), indicating interactions between PS and PP.

### 3.9. Electron Microscopy Scanning

**Figure 13:**
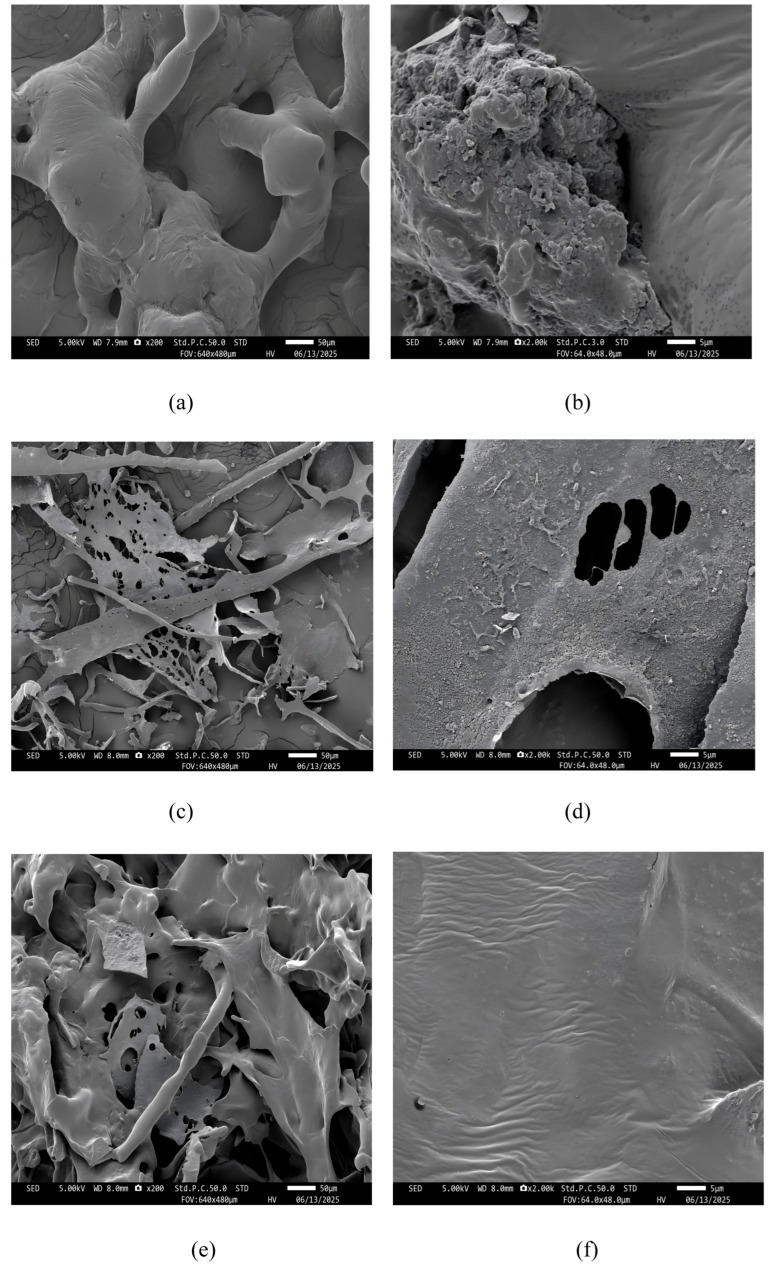
Scanning electron microscopy (SEM) images of *Muzao* polyphenols ((**a**), ×200; (**b**), ×2000), polysaccharides ((**c**), ×200; (**d**), ×2000) and their PS-PP complex ((**e**), ×200; (**f**), ×2000).

Scanning electron microscopy (SEM) is widely used to observe the surface morphology of different compounds. As shown in Figure 13, MPS has a rough, uneven surface with rod-like and irregular spherical protrusions (a,b). PP exhibits a flaky, porous, compact structure (c,d), while the PS-PP complex surface combines polyphenol protrusions with polysaccharide textures, differing from both PP and PS surfaces (e,f). At low magnification, PS-PP appears as a combination of spherical protrusions and porous sheets. At 2k high magnification, its surface texture becomes more complex, with dense wrinkles and irregular undulations.

## 4. Discussion

This study successfully prepared the polysaccharide–polyphenol complex (PS-PP) from *Muzao*, optimized its purification process, evaluated its inhibitory activity against α-glucosidase and α-amylase, and conducted structural characterization. To better elucidate the research findings and highlight their contributions, the key results are compared with previous literature as follows. The adsorption performance of macroporous resins is influenced by polarity, surface characteristics, pore structure, and solubility [48], with polar resins generally exhibiting better adsorption effects than non-polar ones. Although D101 is a non-polar resin, its significantly higher adsorption and desorption rates compared to the other resins may be attributed to its larger average pore size and greater specific surface area than those of HPD100 and AB-8 resins. This adsorption–desorption behavior is consistent with that reported in previous studies on walnut diaphragm polyphenol purification using D101 resin [49]. During the 0–4 h period of the static adsorption process of PP, the resin’s adsorption rate increased rapidly, demonstrating significant adsorption efficiency. This occurred because the resin was not fully saturated, exposing numerous adsorption sites and enabling concentrated PP adsorption [50]. The loading concentration determines the extent of contact between polyphenols and resin, thereby influencing adsorption efficiency. Within a specific concentration range, higher concentrations increase the contact area between polyphenol molecules and the resin, thereby boosting the resin’s adsorption capacity [34]. The adsorption effect of the resin decreased after the concentration reached 0.25 mg/mL. This may be because the viscosity is relatively high at this concentration, hindering the diffusion of molecules and increasing the competition between impurities and the resin, which results in a decrease in the adsorption rate of polyphenols [34]. The effect of pH on adsorption performance is closely related to the ionization state of phenolic hydroxyl groups in MPP. Under acidic conditions (pH ≤ 4), the ionization of phenolic hydroxyl groups is inhibited, and MPP exists mainly in molecular form, which is more easily adsorbed by D101 resin. With the increase in pH value, the ionization degree of phenolic hydroxyl groups increases, and MPP exists in ionic form, which reduces its affinity with the resin, thus leading to the decrease in adsorption rate [51]. Polyphenols are highly soluble in ethanol solutions, so ethanol concentration is a critical factor affecting their desorption efficiency. When the ethanol concentration was 60%, polyphenols had polarity similar to that of the resin; as the volume fraction of the eluent increased, the interfacial forces between them diminished, thereby enhancing the desorption capacity [50]. However, when the ethanol concentration exceeded 60%, the desorption rate declined due to the elution of non-polyphenolic substances, which reduced polyphenol purity; meanwhile, saturated adsorption sites on the resin hindered further polyphenol dissolution [52,53]. The resin’s adsorption capacity increases with sample volume within a specific volume range. However, reaching saturation levels leads to polyphenol waste in the sample solution and complicates resin regeneration. Conversely, insufficient sample volume reduces experimental efficiency. The optimal loading volume (desorption capacity) is reached when the polyphenol concentration in the eluate is 10% (or 1/10) of that in the loading solution. This critical point is recognized as the resin’s polyphenol adsorption leakage threshold [54]. In the dynamic adsorption process of macroporous resin, the liquid flow rate is a critical factor that affects the efficiency of both adsorption and desorption. This is because excessively high flow rates result in a shorter retention time for polyphenols within the chromatographic column, which prevents the resin from reaching saturation, causing most polyphenols to elute with the solution and compromising adsorption efficiency [50]. Conversely, slower loading flow rates ensure thorough contact between the polyphenols in the loading solution and the resin, which results in a lower polyphenol concentration in the eluate per unit volume and delayed leakage point occurrence [53].

T2DM is the most common type of diabetes, and its pathogenesis is mainly associated with environmental factor-induced pancreatic β-cell dysfunction, which further leads to insulin resistance and related complications [55]. α-Glucosidase can hydrolyze glycosidic bonds to generate glucose, and its inhibition can reduce blood glucose levels and protect pancreatic function [56]; α-amylase decomposes starch chains into glucose and maltose, making both enzymes key targets for postprandial blood glucose regulation [57]. The purified *Muzao* PS and PP exhibited higher enzyme inhibitory activities than crude extracts, which was attributed to the removal of impurities and the enrichment of active components, enhancing the binding affinity between active substances and enzyme active sites. This provides a theoretical basis for the subsequent complexation of purified polysaccharides and polyphenols to improve hypoglycemic activity. The enhanced enzyme inhibitory activity of the PS-PP complex compared with individual PS and PP may be attributed to the synergistic effect between polysaccharides and polyphenols. This finding is consistent with the results of previous studies [25,26,58]. The higher inhibitory potency of PS-PP highlights its great potential as a natural hypoglycemic agent for the development of functional foods for type 2 diabetes, and the mechanistic basis for this superior synergism is distinct from earlier studies: Within the 0.05–0.25 mg/mL range, the inhibitory capacity for both enzymes showed a sharp increase, which may be attributed to optimized molecular collision frequency and precise binding site matching at elevated concentrations, enabling the formation of high-yield, stable complexes. Continued concentration increases might reduce efficacy due to excessive aggregation and non-specific binding caused by high molecular loading, which impairs the stability and functional activity of the complex and limits the further improvement of inhibitory efficiency [59].

As evidenced in Section 3.3.2, MPP has stronger α-glucosidase inhibitory activity than PS; the change in α-glucosidase inhibitory rate with mass ratio may be related to the content balance of PS and PP. When the PP content decreases and the PS content increases at a ratio of 3:2, the inhibitory capacity may be compromised, likely due to a reduced PP concentration in the complex, diminishing the original functionality of PP and consequently lowering inhibitory activity. Between 3:2 and 2:1, the complex’s inhibitory effect on α-amylase activity showed an upward trend, possibly because, at 3:2, the PS content was too high and PP was fully bound to the PS active sites, resulting in weaker PP function [60]. When the mass ratio of PS to PP was 2:1, the complex achieved the optimal inhibitory activity; this is a *Muzao*-specific mass ratio effect that has not been reported in other jujube or fruit-based PS-PP complexes. At pH = 4, the inhibitory activity of both enzymes reaches its maximum. This may be due to the peak electrostatic interaction between phenolic hydroxyl groups and polysaccharide carboxyl groups, enabling stable complex formation and optimal functional performance under acidic conditions [61,62]. At pH = 2, strong acids may disrupt glycosidic bonds in polysaccharides and benzene ring structures in polyphenols, reducing their binding capacity and preventing complex formation, which ultimately leads to a significant decrease in inhibitory effect. This supplements the research on the acid stability of jujube-derived PS-PP complexes that was lacking in earlier studies.

Gallic acid, caffeic acid, and protocatechuic acid were previously reported to be contained in jujube polyphenols [63], but were not detected in this study. This deficiency may be caused by changes in jujube varieties, maturity stages, extraction methods, or other factors [64]. In addition, Liu et al. [65] analyzed the changes in polyphenol content at different stages of red date development and found that flavonol and flavonoid content varied over time. Other studies have shown that heat treatment (ranging from 30 °C to 90 °C) increases the tannic acid content of persimmon peel [66]. This study revealed that *Muzao* has a distinctive polyphenol profile, with flavonoids accounting for 42.9% of the total identified phenolic compounds—a proportion significantly higher than that of *Dongzao* (*Ziziphus jujuba* Mill. cv. *Dongzao*) (28.3%) and *Junzao* (*Ziziphus jujuba* Mill. cv. *Junzao*) (31.7%) [67]. This high flavonoid content endows *Muzao* PS-PP complexes with more potent antioxidant and hypoglycemic activities.

The type, ratio, and bonding mode of monosaccharides are key factors affecting the biological activity and physicochemical properties of polysaccharides, which is crucial for clarifying the structure–activity relationship of jujube polysaccharides [68]. Previous studies have shown that jujube polysaccharides also contain the above nine monosaccharides [69]; the same monosaccharide types were identified in *Muzao* polysaccharides in this study, yet their molar ratio (Ara: Gal: Rha = 35.68:31.00:13.58) differed significantly from that of other jujube cultivars (Ara: Gal: Rha = 26.5:35.2:10.8), which could be attributed to differences in cultivation conditions, extraction and purification methods, or detection techniques [70]. This specific monosaccharide ratio is the structural basis for the stronger binding ability of *Muzao* polysaccharides to polyphenols, which is a key reason for the superior synergistic effect of PS-PP complexes.

The laser scattering (LS) signal is proportional to the molecular size and weight of the substance, while the refractive index (RI) signal is associated with the type, concentration, and molecular weight of the detected compound. The distinct PP and PS signal peaks in the elution profile of MPS-PP (Figure 11c) indicate the formation of the PS-PP complex. Changes in Mw are a key indicator of conjugation reactions; the reduced Mw of PS-PP compared to PS also supports the successful coupling of PS and PP, and this finding is consistent with previous studies on chitosan-proanthocyanidin and arabinoglucan-catechin conjugates [71,72,73,74]. This Mw reduction is closely related to the enhanced enzyme inhibitory activity. Specifically, the Mw reduction in *Muzao* PS-PP complexes leads to a smaller molecular size, which reduces steric hindrance and improves the accessibility of the complex to the active sites of α-glucosidase and α-amylase. This demonstrates that the optimized conjugation process in this study induced more stable intermolecular interactions (hydrogen bonding and hydrophobic interactions), rather than the weak physical interactions present in simply mixed complexes.

The absorption peak at 701 cm^−1^ in PS (shifted to 699 cm^−1^ in the complex) suggests the presence of amino sugars or modified amino groups [64,75,76,77]. The increased intensity of the 2936 cm^−1^ peak in the complex may be attributed to end-group disintegration [78], and the enhanced or vanished peaks (2936, 1732, 865, 885, 834 cm^−1^) confirm the formation of stable hydrogen bonds between PS and PP. These hydrogen bonds enhance the structural stability of the PS-PP complex and promote the exposure of active groups (e.g., phenolic hydroxyl groups in PP and hydroxyl groups in PS) that can bind to the active sites of α-glucosidase and α-amylase, thereby strengthening the binding affinity between the complex and the two enzymes and improving enzyme inhibition activity. The peaks at 1625, 1523, 1418, and 1245 cm^−1^ are associated with the benzene ring framework (C=C) of polyphenols and aromatic/polysaccharide C-H vibrations [79]. The absence of new peaks in the 1200–1000 cm^−1^ range may result from overlapping stretching vibrations of C-O-C and C-O glycosidic bonds in PS, and C-O bonds in the benzopyran structure of polyphenols—all of which fall within this wavenumber range [80,81], which is a common structural feature of plant PS-PP complexes but was first confirmed in *Muzao*-derived complexes. The multiple peaks in the 750–900 cm^−1^ range for PP and the complex indicate the presence of benzene ring frameworks (C=C) and substituted structures with varying substituents [82]. These spectral changes confirm that PS and PP interacted successfully, altering their original spectral features [72], which provide additional binding sites for the interaction of the complex with the two enzymes, thereby further enhancing the enzyme inhibitory effect.

The flaky, porous structure of MPS is similar to that observed in previous SEM analyses of garlic polysaccharides [83], which may be caused by intermolecular repulsion leading to sheet-like structural fractures and increased surface porosity [75]. These surface variations stem from interactions between polysaccharides and polyphenols. On the one hand, hydrogen bonds form between the hydroxyl (-OH) groups of the polyphenol and polysaccharide molecules, creating stable yet irregular structures due to the strong intermolecular hydrogen bonding. On the other hand, the hydrophobic aromatic rings of the polyphenol molecules can intercalate into the polysaccharide network to form a tighter structural arrangement [72,80]. This demonstrates that the optimized conjugation process employed in this study induced a more profound structural rearrangement of *Muzao* polysaccharides and polyphenols, which also serves as the structural basis for the superior synergistic hypoglycemic activity of the complex. The tighter and more irregular surface morphology increases the specific surface area of the PS-PP complex, providing more binding sites for the two enzymes and enhancing the inhibition capacity of the complex for the two enzymes. This demonstrates that the optimized conjugation process employed in this study induced a more profound structural rearrangement of *Muzao* polysaccharides and polyphenols, and the structural changes (reduced Mw, stable hydrogen bonding, altered surface morphology) collectively form the structural basis for the superior synergistic hypoglycemic activity of the complex—establishing a clear mechanistic link between structural characteristics and enzyme inhibitory function.

Despite these valuable findings, this study has several limitations. The IC_50_ values of the PS-PP complex from in vitro assays only reflect its enzyme inhibitory activity under controlled in vitro conditions, and cannot represent its bioavailability, digestive stability, in vivo metabolism, or actual therapeutic efficacy in organisms. In vitro experiments failed to simulate key in vivo processes including intestinal flora metabolism and insulin signaling pathway regulation, making it difficult to directly reflect the complex’s actual efficacy in animals or humans. Additionally, the relationship between the structural characteristics of the PS-PP complex and its hypoglycemic activity has not been thoroughly analyzed, with relatively single evaluation indicators.

Future research can establish an animal hypoglycemic model to investigate the complex’s effects on blood glucose levels, insulin concentrations, and liver glycogen content. Combined with cell models, the complex’s regulatory effects on the insulin signaling pathway and key protein expression can be explored to clarify the correlation between in vitro activity and in vivo efficacy. Using advanced characterization techniques (e.g., NMR, X-ray crystallography), the binding sites and spatial conformations of polyphenols and polysaccharides can be elucidated to reveal the molecular-level structure-activity relationship. Based on the complex’s physicochemical and active properties, functional foods (e.g., compressed tablets, candies) or dietary supplements can be developed, and multi-component hypoglycemic products formulated to expand application scenarios, such as special diets for diabetic patients and functional feeds.

## 5. Conclusions

This study investigated the effects of concentration, pH, and mass ratio on the hypoglycemic activity of *Muzao*-derived polysaccharide–polyphenol (PS-PP) complex and explored the interaction between PS and PP via molecular weight, FT-IR, and SEM analyses. The results showed that PS-PP complex significantly enhanced the inhibitory activity of polysaccharides against α-glucosidase and α-amylase, with maximum inhibitory rates of 83.5% and 76.52%, respectively; this enhancement was dependent on environmental factors and the mass ratio of polyphenols in the complex. FT-IR and SEM characterizations revealed that hydrogen bonding between PS and PP altered the structure, molecular weight distribution, and surface morphology of natural polysaccharides, thereby improving polyphenol stability and bioactivity and overcoming their poor bioavailability. These findings clarify the physical factors affecting PS-PP complex and their interaction mechanisms, providing a promising strategy to enhance the structure, stability, and hypoglycemic activity of polyphenols through polysaccharide binding. Overall, the results offer theoretical support for exploring new PS-PP complex sources, developing anti-diabetic drugs and functional foods, and increasing the added value of *Muzao*.

## Figures and Tables

**Table 1:** Comparison of static adsorption and desorption performance of six macroporous resins on polyphenols from *Muzao*.

Resin Type	Polar	Average Pore Size (nm)	Specific Surface Area (m^2^/g)	Adsorption Rate (%)	Desorption Rate (%)
D101	Non polar	9~10	500~700	86.75 ± 0.93 ^a^	94.41 ± 0.76 ^a^
XAD2	Non polar	20~50	300~430	71.44 ± 1.36 ^e^	78.43 ± 1.23 ^e^
HPD-100	Non polar	8~9	650~700	82.54 ± 0.89 ^b^	88.71 ± 0.74 ^b^
AB-8	Weak polar	12~16	480~520	80.01 ± 1.50 ^c^	86.71 ± 1.21 ^c^
ADS-17	Medium polar	25~30	90~150	62.41 ± 1.22 ^f^	69.31 ± 0.62 ^f^
DA201	Polarity	10~13	150~200	76.00 ± 2.03 ^d^	84.03 ± 1.59 ^d^

Note: Values are expressed as the mean ± SD (*n* = 3). Different lowercase letters in the same column indicate significant differences based on Duncan’s multiple range test (*p* ≤ 0.05).

**Table 2:** Phenolic compounds identified in purified *Muzao* polyphenols by liquid chromatography–mass spectrometry (LC-MS).

Number	Substance	Formula	Precursor (*m*/*z*)	Reference (*m*/*z*)	Model Masses (Da)
1	Gallic acid	C_7_H_6_O_5_	171.0278	171.0288	170.0215
2	Epicatechin	C_15_H_14_O_6_	290.0790	291.0860	290.0790
3	Quercetin	C_15_H_10_O_7_	303.0486	303.0499	302.0427
4	Vanillic acid	C_8_H_8_O_4_	169.0482	169.0495	168.0422
5	Protocatechuic acid	C_7_H_6_O_4_	153.0181	153.0182	154.0266
6	Mangiferitin	C_13_H_8_O_6_	261.0396	261.0394	260.0321
7	Quercitrin	C_21_H_20_O_11_	449.1057	449.1078	448.1006
8	Isoquercitin	C_21_H_20_O_12_	465.0996	465.1000	464.0955
9	Catechin gallate	C_23_H_20_O_11_	473.1072	473.1079	472.1001
10	Kaempferol	C_15_H_10_O_6_	287.0548	287.0550	286.0477
11	Nobiletin	C_21_H_22_O_8_	403.1362	403.1387	402.1314
12	Procyanidin A1	C_30_H_24_0_12_	577.1315	577.1341	576.1268
13	Myricetin	C_15_H_10_O_8_	319.0421	319.0440	318.0375
14	Neoeriocitrin	C_27_H_32_O_15_	597.1806	597.1810	596.1641
15	p-Hydroxyphenylacetic acid	C_8_H_8_O_3_	153.0531	153.0550	152.0473
16	3-O-Methylquercetin	C_16_H_12_O_7_	315.0501	315.0500	316.0583
17	Trihydroxyethylrutin	C_33_H_42_O_19_	743.2472	743.2399	742.2320
18	Quercetin 3-galactoside	C_21_H_20_O_12_	463.0882	463.0882	464.0955
19	Quercetin 3,4′-diglucoside	C_27_H_30_O_17_	625.1364	625.1410	626.1483
20	Isorhamnetin	C_16_H_12_O_7_	317.0634	317.0660	316.0583
21	Dehydrodiisoeugenol	C_20_H_22_O_4_	327.1552	327.1591	326.1518
22	Chrysoeriol	C_12_H_16_O_6_	301.0689	301.0710	300.0634
23	Luteolin	C_15_H_10_O_6_	287.0522	287.0550	286.0477
24	Butin	C_15_H_12_O_5_	273.0730	273.0757	272.0685
25	Resveratrol	C_14_H_12_O_3_	229.0825	229.0859	228.0786
26	4-Hydroxycinnamic acid	C_9_H_8_O_3_	165.0529	165.0530	164.0473
27	Salicyluric acid	C_9_H_9_NO_4_	196.0600	196.0600	195.0532
28	Gingerol	C_17_H_26_O_4_	295.1870	295.1800	294.1831
29	Coniferin	C_16_H_22_O_8_	343.1389	343.1390	342.1315
30	Rhusflavanone	C_30_H_22_O_10_	543.1273	543.1286	542.1213
31	Dalbergioidin	C_15_H_12_O6	289.0668	289.0707	288.0634
32	3-Feruloylquinic acid	C_17_H_20_O_9_	369.1202	369.1182	368.1107
33	trans-p-coumaric acid-4-O-glucopyranoside	C_15_H_18_O_8_	327.1045	327.1070	326.1002
34	Isoferulic acid	C_10_H_10_O_4_	193.0511	193.0509	194.0579
35	Cyanidin 3-rutinoside	C_27_H_31_O_15_	596.1652	596.1730	595.1663
36	Quercetin 3-(2R-apiosylrutinoside)	C_32_H_38_O_20_	743.1984	743.2070	742.1957
37	Astragalin	C_21_H_20_O_11_	447.0889	447.0933	448.1006
38	Procyanidin B2	C_30_H_26_O_12_	577.1326	577.1351	578.1424
39	Procyanidin C1	C_45_H_38_O_18_	865.2017	865.1986	866.2058
40	3′-Galloylquercitrin	C_28_H_24_O_15_	601.1147	601.1188	600.1115
41	Luteolin 7-methyl ether	C_16_H_12_O_6_	299.0539	299.0561	300.0634
42	Myricetin 3,3′-digalactoside	C_27_H_30_O_18_	641.1278	641.1357	642.1432
43	Myricetin-3-O-xyloside	C_20_H_18_O_12_	449.0684	449.0670	450.0798
44	Myricetin 3-glucoside	C_21_H_20_O_13_	479.0844	479.0831	480.0904
45	Kaempferol-3-O-rutinoside	C_27_H_30_O_15_	593.1519	593.1512	594.1585
46	Kaempferol 3-sophoroside 7-glucoside	C_33_H_40_O_21_	771.1988	771.1989	772.2062
47	Demethylnobiletin	C_20_H_20_O_8_	387.1129	387.1086	388.1158
48	Kaempferol 3-gentiobioside 7-rhamnoside	C_33_H_40_O_20_	755.2034	755.2036	756.2113
49	Kaempferol-3-O-glucoside-6′-p-coumaroyl	C_30_H_26_O_13_	593.1262	593.1301	594.1373

## Data Availability

The original contributions presented in the study are included in the article/Supplementary Materials, and further inquiries can be directed to the corresponding author.

## Supplementary Materials

The following supporting information can be downloaded at https://www.mdpi.com/article/10.3390/foods15030552/s1. Figure S1. Standard calibration curves for polysaccharide and polyphenol quantification. (a) Poly-saccharide calibration curve (0–100 μg/mL, y = 0.0056x + 0.087, R^2^ = 0.9993); (b) Polyphenol calibration curve (0–140 μg/mL, y = 0.0048x + 0.092, R^2^ = 0.9996). Table S1. Details of commonly used instruments.
