# Supplementary material for: Preparation, Structural Characterization, and Synergistic Hypoglycemic Effect of Jujube Polysaccharide–Polyphenol Complex
Source: Foods. 2026 Feb 4;15(3):552. doi: 10.3390/foods15030552 (PMC12896497; doi:10.3390/foods15030552)
Supplement: Supplementary file 1 [file foods-15-00552-s001.zip › foods-4082091-supplementary.pdf]

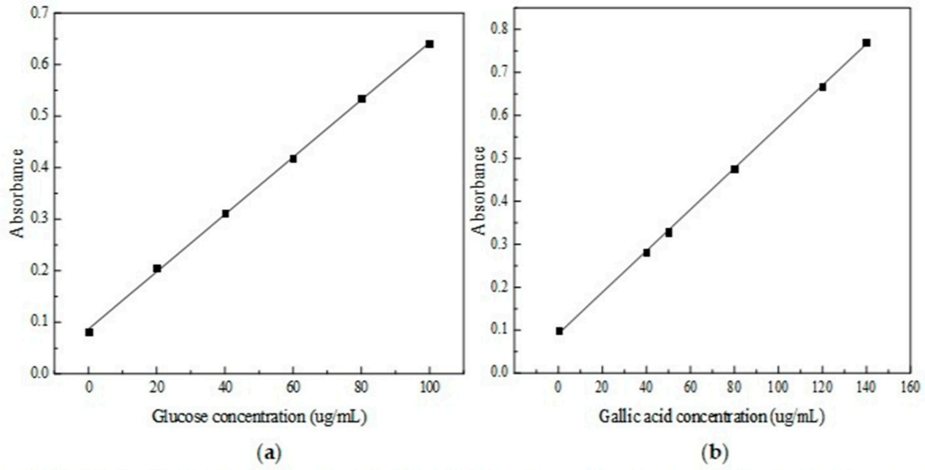

Figure S1. Standard calibration curves for polysaccharide and polyphenol quantification. (a) Polysaccharide calibration curve (0–100 μg/mL, $y = 0.0056x + 0.087$, $R^2 = 0.9993$); (b) Polyphenol calibration curve (0–140 μg/mL, $y = 0.0048x + 0.092$, $R^2 = 0.9996$).

Table S1. Details of commonly used instruments.

Instrument Name	Model	Manufacturer	Country
Electric Thermostatic Water Bath	DZKW-4	Beijing Zhongxing Weiye Century Instrument Co., Ltd.	China
Numerical Control Ultrasonic Cleaner	KQ5200DE	Kunshan Ultrasonic Instrument Co., Ltd.	China
Circulating Water Vacuum Pump	SHZ-III	Shanghai Yalong Biochemical Instrument Co., Ltd.	China
pH Meter	PHS-3C	INESA Scientific Instrument Co., Ltd.	China
Electric Blast Drying Oven	Model 101	Shanghai Jianyi Instrument Co., Ltd.	China
Centrifuge	TDL-5-A	Shanghai Anting Scientific Instrument Factory	China
Constant Flow Pump	HL-2B	Shanghai Jiapeng Technology Co., Ltd.	China
Crusher		Zhejiang Hongjingtian Industry and Trade Co., Ltd.	China
Vacuum Freeze Dryer		Ningbo Xinzhi Biotechnology Co., Ltd.	China
Microplate Reader		Shanghai Senpushi Biotechnology Co., Ltd.	China
UV-Visible Spectrophotometer	TU-1810PC	Beijing Purkinje General Instrument Co., Ltd.	China
